# Systematic review: conservative treatments for secondary lymphedema

**DOI:** 10.1186/1471-2407-12-6

**Published:** 2012-01-04

**Authors:** Mark Oremus, Ian Dayes, Kathryn Walker, Parminder Raina

**Affiliations:** 1 Department of Clinical Epidemiology and Biostatistics, McMaster University, Hamilton, Ontario, Canada; 2 McMaster Evidence-based Practice Centre, McMaster University, 1280 Main Street West, DTC-310, Hamilton, Ontario, L8S 4K1, Canada; 3 Department of Oncology, McMaster University, Hamilton, Ontario, Canada

## Abstract

**Background:**

Several conservative (i.e., nonpharmacologic, nonsurgical) treatments exist for secondary lymphedema. The optimal treatment is unknown. We examined the effectiveness of conservative treatments for secondary lymphedema, as well as harms related to these treatments.

**Methods:**

We searched MEDLINE^®^, EMBASE^®^, Cochrane Central Register of Controlled Trials^®^, AMED, and CINAHL from 1990 to January 19, 2010. We obtained English- and non-English-language randomized controlled trials or observational studies (with comparison groups) that reported primary effectiveness data on conservative treatments for secondary lymphedema. For English-language studies, we extracted data in tabular form and summarized the tables descriptively. For non-English-language studies, we summarized the results descriptively and discussed similarities with the English-language studies.

**Results:**

Thirty-six English-language and eight non-English-language studies were included in the review. Most of these studies involved upper-limb lymphedema secondary to breast cancer. Despite lymphedema's chronicity, lengths of follow-up in most studies were under 6 months. Many trial reports contained inadequate descriptions of randomization, blinding, and methods to assess harms. Most observational studies did not control for confounding. Many studies showed that active treatments reduced the size of lymphatic limbs, although extensive between-study heterogeneity in areas such as treatment comparisons and protocols, and outcome measures, prevented us from assessing whether any one treatment was superior. This heterogeneity also precluded us from statistically pooling results. Harms were rare (< 1% incidence) and mostly minor (e.g., headache, arm pain).

**Conclusions:**

The literature contains no evidence to suggest the most effective treatment for secondary lymphedema. Harms are few and unlikely to cause major clinical problems.

## Background

Secondary lymphedema (SE) is an acquired condition resulting from disease, trauma, or an iatrogenic process such as surgery or radiation that damages the lymphatic system [1,2]. Clinically, SE may present as edema [3].

Globally, the major cause of SE is lymphatic filariasis resulting from infection with the nematode *Wusheria Bancrofti*. In the United States (U.S.), the most common cause of SE is treatment for malignancy (i.e., surgery, radiation) [4], especially breast cancer. SE incidence rates following mastectomy range from 24% to 49%, with lower rates of 4% to 28% following lumpectomy [1]. The literature is bereft of reliable prevalence estimates, although some suggest approximately 10 million persons in the U.S. have SE http://www.shlnews.org/?p=67.

Several types of conservative therapy exist to treat SE. Compression techniques, including multilayer bandaging, and pressure garments are thought to restore hydrostatic pressure and improve lymph flow in affected limbs [5]. Manual lymphatic drainage (MLD), a form of massage, is administered using light strokes to direct lymph flow from blocked to open lymphatics [5-7]. Exercise helps increase lymph flow via muscle contraction around the lymphatics [8]. Complex (or complete) decongestive therapy (CDT) includes MLD, limb compression with low stretch bandages, skin care, and exercise. The intent of CDT is to decrease fluid in affected limbs, prevent infection, and improve tissue integrity [5,9]. Dieting (e.g., low-fat diet) is also used as a conservative therapy for SE.

Mechanical treatments for SE include intermittent pneumatic compression (IPC) devices and low-level laser therapy (LLLT). IPC devices are pneumatic cuffs connected to pumps that mimic the naturally occurring muscle pump effect of muscles contracting around peripheral lymphatics [10]. LLLT employs low intensity laser waves and appears to encourage formation of lymphatic vessels, promote lymph flow, and stimulate immune systems [11,12].

This systematic review is based on a peer-reviewed technology report [13] commissioned by the Agency for Healthcare Research and Quality (AHRQ) and the Centers for Medicare and Medicaid Services (CMS). A copy of the technology report is available on the AHRQ website http://www.cms.gov/determinationprocess/downloads/id66aTA.pdf. The technology report served as background material for a Medicare Evidence Development & Coverage Advisory Committee (MEDCAC) Meeting held in November 2009. One purpose of the meeting was to discuss the available evidence for treatment methods in SE.

This review addresses two key questions:

1. How effective are conservative treatments for SE in pediatric or adult populations who developed SE following any type of illness except filariasis infection?

2. What harms are associated with conservative treatments for SE?

## Methods

### Data sources and selection

We searched MEDLINE^®^, EMBASE^®^, Cochrane Central Register of Controlled Trials^®^, AMED, and CINAHL from 1990 to January 19, 2010. We exploded the subject heading 'lymphedema' and searched it as a textword ('lymphedema' or 'lymphoedema'). The complete literature search strategy is depicted in Additional file 1 Methods S1. We initially searched the English-language literature and later searched the non-English literature following recommendations of persons who peer reviewed our technology report [13]. The purpose of exploring non-English studies was to assess whether they contained information to supplement the English-language studies. We also searched the reference lists of extracted studies and previously published systematic reviews [1,12,14-16].

### Criteria for considering studies for this review

We included studies provided they were randomized controlled trials (RCTs) or observational studies with comparison groups (e.g., cohort, case control). We also included studies of pediatric and adult patients who received treatment for SE following any form of illness except filariasis infection. We excluded case series, case reports, narrative and systematic reviews, editorials, comments, letters, opinion pieces, abstracts, conference proceedings, and animal experiments. We also excluded studies involving pharmacologic or surgical treatments for SE.

Trained raters independently applied the inclusion and exclusion criteria to the articles retrieved in the literature search. The criteria were applied at three levels of screening: I-title and abstract first review; II-title and abstract second review; III-full text. We extracted data from articles that passed full text screening. Raters managed the screening process electronically using standardized screening forms and Distiller SR systematic review software (Evidence Partners, Ottawa, Canada).

### Methodological quality assessment

Two raters independently assessed the quality of the extracted English-language articles. Raters used the eight-point Jadad scale for RCTs [17,18] and the Newcastle-Ottawa Scale (NOS) [19] for observational studies. The overall quality of each extracted article was rated 'good', 'fair', or 'poor' in accordance with the recommendations outlined in the AHRQ's methods guide for systematic reviews [20].

Issues of methodological quality often preclude the inclusion of observational studies in systematic reviews. However, observational studies may be included to help overcome evidence gaps in RCTs, especially in the assessment of harms [20].

### Data extraction

A meta analysis was infeasible because the extracted studies exhibited substantial clinical and methodological heterogeneity. Therefore, we used a descriptive approach to answer the key questions. This approach involved extracting English-language data into tables and developing written summaries of the English and non-English evidence.

For English-language articles, we extracted data on study design, type of treatment, sample size, cause of SE, definition of SE, study inclusion/exclusion criteria, and outcome data. While we did not extract data from the non-English articles, we summarized the main contents of these articles in writing and compared them to the extracted English-language articles.

### Role of the funding source

The McMaster University Evidence-based Practice Centre researched and wrote the initial technology report under contract with the AHRQ, which gave us permission to publish this manuscript. The AHRQ and CMS had no role in the literature search, data analysis, study conduct, manuscript preparation, or interpretation of results.

## Results

**Figure 1:**
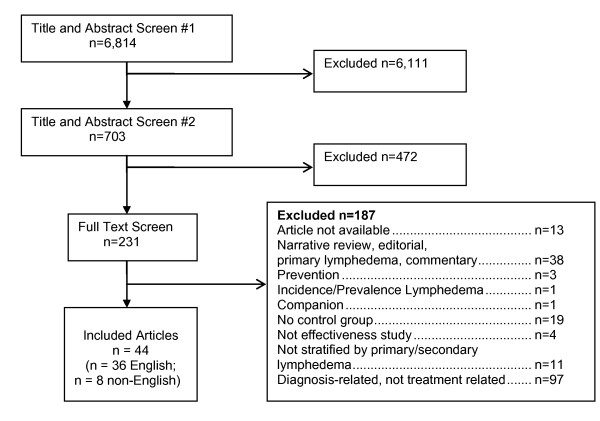
**Study flow diagram**.

Figure 1 depicts the flow of studies through screening. Thirty-six English-language and eight non-English-language studies passed screening. Table 1 contains basic information on the English-language studies; Table 2 shows extracted English-language data relevant to answering the two key questions listed above.

**Table 1:** Basic study data

Study (Quality^†^)	Sample Size (Treatment + Control)	1) Cause of SE 2) Definition of SE	1) Time of SE Onset 2) Time of Tx initiation 3) Criteria to Start/stop Tx	Other Inclusion/Exclusion Criteria
**RCT**				

Andersen 2000 [21] Denmark Fair (4)	20 + 22	1) BCa Tx 2)≥ 200 ml volume or ≥ 2 cm circumference difference between arms	1) After surgery 2)≥ 4 month post-BCa Tx 3) SE Dx/NR	Exclusion: - bilateral BCa - SE Tx < 3 months - BCa recurrence - severe SE (volume difference > 30%)

Bertelli 1991 [22] Italy Fair (4)	37 + 37	1) BCa Tx 2)> 10 cm and < 20 cm circumference difference between arms (mild SE)	1) Limb circumference ≥ 25% compared to baseline 2) NS	Inclusion: - no metastases or relapse - no Tx ≤ 6 months - no lymphangitis Exclusion: - wearing cardiac stimulator - currently receiving CT or RT

Bialoszewski 2009 [23] Poland Poor (3)	12 + 12	1) Lower extremity SE post-leg lengthening surgery 2) Physical examination and radiographic images to Dx SE	1) Following leg lengthening surgery 2) Post-surgery 3) Lower extremity SE/NR	Inclusion: -age 15-40 years

Carati 2003 [11] Australia Good (8)	37 + 27	1) BCa Tx 2)> 200 ml volume or ≥ 2 cm circumference difference between arms	1) NR 2) NR 3) SE Dx/NR	Inclusion: - female Exclusion: - co-morbidities present - significant change ≤ 3 months - unable to manipulate arm - primary SE

Damstra 2009 [24] Netherlands Good (6)	18 + 18	1) BCa Tx 2) Moderate to severe SE (ISL definition)	1) 3-50 month post-surgery 2)≥ 12 month post-surgery 3) SE Dx/NR	Inclusion: -female -> 18 years -12 months post BCa Tx without reoccurrence Exclusion: -allergy to materials -systemic diseases -arterial/venous disease

Didem 2005 [25] Turkey Fair (5)	27 + 26	1) BCa Tx 2) Arm circumference difference 2-5 cm	1)> 1 year after surgery 2) 3 year post-surgery 3) SE Dx/NR	Inclusion: - SE ≥ 1 year Exclusion: - psychiatric illness - pain in axillary region - cardiac disease - uncontrolled hypertension - malignancy

Dini 1998 [26] Italy Fair (5)	40 + 40	1) BCa Tx 2) Arm circumference difference of 2-5 cm	1)< 1 year 2)> 1 year after SE 3) SE Dx/difference in circumference > 10 cm in affected vs. unaffected limb/occurrence of harms	Inclusion: - SE ≥ 1 year - no lymphangitis, no evidence of local or distant relapse, no other serious or psychiatric illness Exclusion: - prior SE therapy - bilateral breast surgery - bilateral axillary node dissection

Hayes 2009 [27] Australia Good (6)	16 + 16	1) BCa Tx 2) Health professional diagnosis	1) NR 2)≥ 6 month after BCa Tx 3) SE Dx/occurrence of harms	Inclusion: -< 76 years - Unilateral BCa Tx ≥ 6 months ago - able to travel to clinic

Hou 2008 [28] China Poor (3)	15 + 35	1) BCa Tx 2) NR	1) NR 2)> 5 year post-surgery 3) SE Dx/NR	Exclusion: - radiotherapy

Irdesel 2007 [29] Turkey Fair (5)	10 + 11	1) BCa Tx 2) NR	1) 3-60 month 2)> 4 month post-BCa surgery 3) SE Dx/NR	Exclusion: -BCa operation < 4 months ago -recurrence or bilateral BCa -stage 4 BCa -elephantiasis -congestive heart failure -deep vein thrombosis -acute infection

Jahr 2008 [30] Germany Fair (5)	11 + 10	1) BCa Tx 2) NR	1) NR 2) ~4 year post-surgery 3) SE Dx/NR	Inclusion: - age 18-80 years - living near study center -≥ 6 weeks since RT Exclusion: - Tx ≤ 3 months ago - acute inflammation - acute thrombosis - heart disease - electronic implant - pregnant - sensitivity to electric fields

Johansson 1998 [31] Sweden Fair (4)	14 + 14	1) BCa Tx 2)> 10% difference in affected vs. unaffected arm	1) Median 9-10.5 month 2) Median 9-10.5 month 3) SE Dx/NR	Exclusion: - previous contralateral breast disease - comorbidity affecting swollen arm - treatment ≤ 6 months (except compression sleeve) - SE resolved during initial compression sleeve use

Kaviani 2006 [32] Iran Fair (5)	4 + 4	1) BCa Tx 2)≥ 2 cm swelling in affected arm	1) 3 month 2) SE ≥ 3 month 3) SE Dx/NR	Inclusion: - no contraindications to laser Exclusion: - metastatic disease

Kessler 2003 [33] Switzerland Poor (3)	11 + 12	1) Ankle surgery 2) Clinically diagnosed post-operative swelling	1) NR 2) 2nd day post-surgery 3) Post-operative swelling/NR	- Age: 18-75 year - good physical condition - no contraindications for lymph drainage

Kozanoglu 2009 [34] Turkey Poor (3)	25 + 25	1) BCa Tx 2) Difference > 2 cm at 3/7 measurement points on limb	1)> 3 month 2) SE > 3 month 3) SE Dx/NR	Inclusion: - arm SE ≥ 3 months Exclusion: - metastases or ongoing RT - cellulitis - venous thrombosis - inflammatory disease - history of severe trauma - photosensitivity - medications that affect electrolyte balance - limitation in UE joints - physical therapy other than skin care - home exercises for SE in past 6 months

Lau 2009 [35] China Good (6)	11 + 10	1) BCa Tx 2) Arm volume difference > 200 ml	1) 22-60 month post-BCa 2) Post-BCa Tx 3) SE Dx/NR	Inclusion: -≥ 18 years - unilateral mastectomy + CT or RT Exclusion: - metastases - history of arm trauma - kidney, heart, or lung disorder - medications that alter body fluids - primary SE of lower limb - decrease shoulder movement - cellulitis ≤ 3 months

Maiya 2008 [36] India Fair (5)	10 + 10	1) BCa Tx 2)≥ 2 cm difference at any 2 points between affected and unaffected limbs	1) NR 2) 3-6 week post-mastectomy 3) SE Dx/NR	Inclusion: - mastectomy or RT completion Exclusion: - primary SE - limb infection

McKenzie 2003 [37] Canada Poor (3)	7 + 7	1) BCa Tx 2) Circumference difference is > 2 cm and < 8 cm	1) NR 2)> 6 month post-cancer Tx 3) SE Dx/NR	Exclusion: - stage III SE - bilateral disease - medications that affect swelling

McNeely 2004 [38] Canada Good (6)	22 + 20	1) BCa Tx 2)≥ 150 ml difference between affected and unaffected arms	1) NR 2) NR 3) SE Dx/NR	Inclusion: - no sleeve use < 4 months -≥ 6 months since SE T XExclusion: - new cancer D X- receiving RT or CT - infection in SE limb - contraindications to T X- uncontrolled hypertension - heart disease - renal insufficiency - venous thrombosis

Pilch 2009 [39] Poland Poor (3)	17 + 9 + 11 + 20	1) BCa Tx 2) NR	1) NR 2) NR 3) SE Dx/NR	Inclusion: -age 39-80 years

Radakovic 1998 [40] Yugoslavia Poor (1)	18 + 18	1) BCa Tx 2) NR	1) NR 2) post-RT 3) SE Dx/NR	Inclusion: - no metastases

Schmitz 2009 [41] U.S. (companion Schmitz [42]) Good (7)	71 + 70	1) BCa Tx 2)≥ 10% volume or circumference difference between affected and unaffected arms	1) NR 2) 1-15 year post-BCa 3) SE Dx/SE exacerbation or cancer recurrence	Inclusion: - 1-15 years since BCa D X- no evidence of cancer - unilateral SE - BMI < 50 kg/m^2^ - not actively trying to lose weight - no medical conditions to limit exercise - no weight lifting ≤ 1 year - removal of at least one lymph node

Shaw 2007 [43] U.K. Fair (5)	11 + 10	1) BCa Tx 2) Affected arm volume ≥ 15% than unaffected arm	1) NR 2)≥ 12 month post-CT or RT 3) SE Dx/completion of therapeutic regimen	Inclusion: - remission - BMI ≥ 25 kg/m^2^

Shaw 2007 [44] U.K. Fair (5)	19 + 17 + 15	1) BCa Tx 2) Affected arm volume ≥ 20% than unaffected arm	1) NR 2)≥ 12 month post-cancer Tx 3) SE Dx/NR	Inclusion: - remission

Sitzia 2002 [45] U.K. Fair (5)	15 + 13	1) BCa Tx 2) Moderate or severe edema (≥ 20%)	1) NR 2) NR 3) SE Dx/NR	Inclusion: -≥ 18 years - no active disease - no Tx except support garment

Szuba 2002 [46] U.S. Fair (4)	12 + 11	1) BCa Tx 2) Affected arm volume ≥ 20% than unaffected arm	1) NR 2)≥ 3 month from BCa Tx 3) SE Dx/NR	Inclusion: -≥ 12 week post T XExclusion: - active infection - recurrence - venous occlusion

Szuba 2002 [47]^††^ U.S. Fair (4)	12 + 13	1) BCa Tx 2) NR	1) NR 2) 1-12 month 3) SE Dx/NR	Inclusion - CDT completed 1-12 months ago Exclusion: - active infection - recurrence - venous occlusion - bilateral SE

Tsai 2009 [48] China Good (6)	20 + 21	1) BCa Tx 2) Affected arm circumference ≥ 2 cm than unaffected arm	1)≥ 3 month post-BCa Tx 2) 4 week after control period 3) SE Dx/NR	Inclusion: - unilateral SE ≥ 3 months Exclusion: - active cancer - use of diuretics or other SE influencing drugs - skin disease - decreased arm motion

Wilburn 2006 [49]^††^ U.S. Good (7)	5 + 5	1) BCa Tx 2) Affected arm volume ≥ 20% than unaffected arm	1) 34 ± 34 month 2) 0-5 month after SE onset 3) SE Dx/NR	Exclusion: - bilateral SE - active cancer or infection - venous obstruction or active thrombophlebitis - pulmonary edema - congestive heart failure - history of pulmonary embolism - contraindications to Tx

Williams 2002 [50]^††^ U.K. Fair (4)	15 + 16	1) BCa Tx 2)> 10% excess volume measured two times	1)> 3 month 2)> 3 month 3) SE Dx/NR	Exclusion: - active cancer - use of edema-influencing drugs

**Observational**				

Balzarini 1993 [51] Italy Good (8)	50 + 100	1) BCa Tx 2)% difference between arms: ≤ 6.5% (mild), 6.5 to 13% (moderate), ≥ 13% (severe)	1) IG: 3-52 month; CG: 5-57 month 2) NR 3) SE Dx/NR	Exclusion: - Tx with regional RT

Berlin 1999 [50] Sweden Fair (6)	28 + 8 +19	1) BCa Tx 2) Affected arm volume ≥ 100 ml than unaffected arm	1) NR 2) NR 3) SE Dx/NR	NR

Brambilla 2006 [53] Italy Good (8)	50 + 15	1) SE due to Kaposi's sarcoma 2) Grade II SE according to ISL	1) NR 2) NR 3) SE Dx/NR	Inclusion: - SE below knee

Frischenschlager 1991 [54] Austria Fair (5)	15 + 15	1) BCa Tx 2) NR	1) ~5 year post-BCa Tx 2) NR 3) SE Dx/NR	Inclusion: -female

Johansson 1999 [55] Sweden Fair (6)	20 + 18	1) BCa Tx 2) Affected arm volume ≥ 10% than unaffected arm	1) NR 2) NR 3) SE Dx/arm swelling resolution	Exclusion: - previous contralateral breast disease - comorbidity affecting swollen arm - treatment ≤ 6 months (except compression sleeve)

Pinell 2007 [56] U.S. Good (7)	16 + 56	1) BCa Tx 2) Affected arm circumference ≥ 2 cm than unaffected arm	1) NR 2) NR 3) SE Dx/NR	Inclusion: - referral to specific clinics

*BCa *Breast Cancer; *BMI *Body Mass Index; *CDT *Complex Decongestive Therapy; *CG *control group; *CT *chemotherapy; *Dx *Diagnosis; *IG *intervention group; *ISL *International Society of Lymphology; mo: months; *NR *Not Reported; *RT *Radiation Therapy; *RCT *Randomized Control Trial; *SE *Secondary Lymphedema; *Tx *Treatment; UE: Upper extremity; *wk *week; *yr *year

^†^Rating (scale score)-RCT Jadad: poor (1-3), fair (4-5), good (6-8); Observational NOS: poor (0-3), fair (4-6), good (7-9)

^††^Crossover RCT (all other RCTs were randomized, parallel group)

**Table 2:** Data extraction for key questions

Study		1) Patient Outcomes		
	**Treatment Protocols**		**Length of Follow-up**	**Tx-related Harms?**

		**2) Results**		

**RCT**				

Andersen	IG: standard care + MLD + self-massage	1) Limb volume, self-reported SE symptoms	12 month	NR

2000 [21]	(standard care: compression garment + exercise instruction + skin care)			

Denmark		2) NS		

	CG: standard care			

Bertelli	IG: sleeve (6 h/day for 6 mths) + IPC (2 cycles of 2 week spaced by 5 week interval	1) Limb circumference ≥ 25% compared to baseline	6 month	NR

1991 [22]				

Italy	CG: sleeve (6 h/day for 6 months)	2) NS		

Bialoszewski	IG: Kinesiotaping (10 days) + standard physiotherapy (not described)	1) Limb circumference	10 days	NR

2009 [23]				

Poland	CG: Lymphatic drainage (1 × /day × 10 days) + standard physiotherapy (not described)	2) Significant reduction in limb circumference with kinesiotaping		

Carati	IG: LLLT (9 sessions, 17 min each, 3 × /week × 3 week; 8-week rest and repeat)	1) Limb circumference and volume	24 month	NR

2003 (11)				

Australia	CG: Sham LLLT	2) NS		

	(9 sessions as above; 8-week rest) + 'active' LLLT (as above)			

Damstra	IG: Low-stretch bandage	1) Limb volume, pain and discomfort	24 h	Patients with high pressure bandages reported more pain and discomfort

2009 [24]				

Netherlands	CG: High-stretch bandage	2) NS (volume)		

Didem	IG: MLD + compression garment + exercise + skin care	1) Limb circumference, range of motion	4 week	NR

2005 [25]				

Turkey	CG: Physiotherapy (bandage + limb elevation + exercises) + compression garment + exercise + skin care	2) Greater decrease in circumference in IG (p < 0.05), NS (ROM)		

Dini	IG: IPC (2 cycles over 2 week; each cycle separated by 5-week interval)	1) Limb circumference	9 week	No harms

1998 [26]				

Italy	CG: Skin care, prophylaxis	2) NS		

Hayes	IG: Aerobic and resistance exercise (12 week)	1) Bioimpedance, perometry	12 week	Swelling (n = 1)

2009 [27]				

Australia	CG: NR	2) NS		

Hou	IG: BMSC + compression garment	1) Limb volume, self-reported pain	52 week	NR

2008 [28]				

China	CG:CDT (MLD + compression therapy + exercise)	2) Volume and pain reductions greater in BMSC group at 52 week (p < 0.05)		

Irdesel	IG: Exercise + compression garment	1) Limb circumference, shoulder range of motion	6 month	NR

2007 [29]				

Turkey	CG: Exercise	2) NS		

Jahr	IG: low-intensity electrostatic field (2-3 ×/week × 4 week) + MLD	1) Visual analogue pain scale	8 week	NR

2008 [30]				

Germany	CG: MLD	2) NS		

Johansson	IG: IPC (2 h/day, 5 days/week for 2 week) + compression garment	1) Limb volume	2.5 year	NR

1998 [31]				

Sweden	CG: Vodder MLD + compression garment	2) NS		

Kaviani	IG: LLLT (3 × /week × 3 week; 8 week interval, then repeat same protocol × 3 week)	1) Limb circumference, visual analogue pain scale	22 week	NR

2006 [32]				

Iran	CG: Sham laser	2) IG more efficacious than CG, but authors report no p-values		

Kessler	IG: Daily physiotherapy exercises + MLD	1) Limb volume	NR	NR

2003 [33]				

Switzerland	CG: Daily physiotherapy exercises	2)% volume reduction-IG vs. CG (6.4% vs. 0.1%, p = 0.011)		

Kozanoglu	IG: Laser (20 min/3 × wk × 4 week) + exercise + skin care	1) Limb circumference, visual analogue pain scale, grip strength	12 month	None

2009 [34]				

Turkey	CG: IPC (2 h at 60 mmHg ×	2) IG improved over CG on circumference (p = 0.02), pain and grip strength (NS)		

	20 sessions over 4 week) + exercise + skin care			

Lau	IG: LLLT 3 × /week for 4 week	1) Limb volume, tissue resistance, DASH score	8 week	NR

2009 [35]				

China	CG: no Tx	2) Mean volume less in IG (p = 0.04), greater tissue resistance in IG at 3 of 4 sites (p < 0.05), DASH (NS)		

Maiya	IG: LLLT (34 min/day for 10 days) + exercise (after laser)	1) Limb circumference, pain scale	10 days	None

2008 [36]				

India	CG: Compression garment (10 days) + exercise	2) IG improved over CG on both outcomes (p < 0.05)		

McKenzie	IG: Stretching, resistance, and aerobic exercise training (3 × /week for 8 week	1) Arm circumference and volume, quality-of-life (SF-36 scale)	8 week	NR

2003 [37]				

Canada	CG: No Tx	2) NS (all outcomes)		

McNeely	IG: MLD (5 days/week × 4 week) + bandaging	1) Limb circumference and volume	4 week	Skin reaction (n = 1), bandage discomfort (n = 1)

2004 [38]				

Canada	CG: Bandaging	2) NS		

Pilch	Different IPC protocols (4 groups):	1) Limb volume	5 week	NR

2009 [39]	-single chamber, 90 s on: 90 sec off			

Poland	-3 chamber, 90 sec on: 90 sec off	2) NS		

	-single chamber, 45 sec on: 15 sec off			

	-3 chamber, 45 sec on: 15 sec off			

Radakovic	IG: IPC (60 min/day × 10 days) + compression bandage	1) Change in arm volume (limb circumference)	10 days	NR

1998 [40]				

Yugoslavia	CG: MLD (30 min/day × 10 days) + compression bandage	2) Circumference reduction greater in IG vs. CG (2.24 cm vs. 0.95; p < 0.05)		

Schmitz	IG: Weight lifting (supervised for13 week, unsupervised for 39 week) + compression garment during exercise	1) Limb volume	12 month	Authors report no serious harms

2009 [41]				

U.S.	CG: 1-year fitness membership and 13 week of supervised instruction (not mandatory)	2) NS		

(companion Schmitz [42])				

Shaw	IG: Dietary advice for weight loss	1) Limb volume	12 week	NR

2007 [43]				

U.K.	CG: Healthy eating booklet + compression garment	2) Significant reduction in SE arm volume IG vs. CG (7% vs. 3% reduction: p < 0.05)		

Shaw	IG: Weight reduction-reduced energy intake OR low fat diet-no reduced energy intake (2 groups)	1) Limb volume	24 week	NR

2007 [44]				

U.K.	CG: No Tx	2) NS		

Sitzia	IG: MLD (40-80 min 5 × wk × 2 week)	1) Limb volume	2 week	NR

2002 [45]				

U.K.	CG: SLD (20 mins 5 × wk × 2 week)	2) NS		

Szuba	IG: MLD (daily, self-administered) + compression garment	1) Limb volume, tonometry, range of motion	6 month	None

2002 [47]				

U.S.	CG: As above + IPC (1 h daily at 40-50 mmHg)	2) Greater mean volume reduction with IPC (p < 0.05); NS (tonometry, range of motion)		

Szuba	IG: MLD (daily) + IPC (30 min at 40-50 mmHg) + compression garment	1) Limb volume, tonometry	30 days	Repetitive headache and small blood pressure increase during IPC (n = 1)

2002 [46]				

U.S.	CG: MLD (daily) + compression garment	2) NS		

	(Maintenance therapy-IG & CG: compression garment + self-administered MLD)			

Tsai	IG: Kinesiotape bandage	1) Limb volume and circumference, symptom severity on visual analogue scales, QoL	3 month	NR

2009 [48]				

China	CG: Short-stretch bandage	2) NS		

	(IG & CG: MLD + IPC + exercise)			

Wilburn	IG: IPC (1 h/day)	1) Limb volume, QoL	42 days	NR

2006 [49]				

U.S.	CG: Self-message (1 h/day) + compression garment	2) Mean volume reduction greater in IG (-208 ml vs.		

		+ 52 ml; p = 0.007), NS (QoL)		

Williams	IG: MLD (daily × 3 week)	1) Limb volume, caliper creep, dermal thickness, QoL	12 week	NR

2002 [50]				

U.K.	CG: SLD (daily × 3 week)	2) NS (limb volume, caliper creep), no intergroup differences reported in article (dermal thickness, QoL)		

**Observational**				

Balzarini	IG: Ultrasound	1) Limb volume	12 month	NR

1993 [51]	(2 cycles at 4 month intervals-one cycle = 10-30 min session)			

Italy		2) NS		

	CG: IPC (6 h/day × 5 days once every 4 month for 12 month)			

Berlin	IG 1: IPC (90-120 mmHg for 20-30 min 2 × /day 5 day/week) + compression garment (25-50 mmHg × 4 week)	1) Limb volume	5 year	NR

1999 [52]				

Sweden	IG 2: IPC (80 mmHg ≥ 20 min/day × 4 week)	2) NS		

	CG: Compression garment (25-50 mmHg × 4 week)			

Brambilla	IG: Compression garment (custom-made, mean pressure = 40 mmHg, worn morning-to-night, changed every 6 month)	1) Limb volume	IG: Mean 66 week	NR

2006 [53]				

Italy	CG: No Tx	2) IG: 30/50 mean reduction = 9.3 ml; 20/50 mean increase = 78.7 ml	CG: Mean 64 week	

		CG: 15/15 mean increase = 29.6 ml		

		(p < 0.0001 between groups)		

Frischenschlager	IG: Psychosocial therapy and exercise (2 h/week × 10 week) + MLD (3 × /day × 10 week) + compression stocking during day	1) Psychic well being and physical complaints scales	10 week	NR

1991 [54]				

Austria	CG: As above except for psychosocial therapy	2) Improved psychic well-being in IG (p = 0.02), NS (physical complaints)		

Johansson	IG: Compression bandage (2 week) + MLD (45 min/day × 5 days in wk 3)	1) Limb volume, body weight, pain/heaviness/tension using visual analogue scales	19 days	NR

1999 [55]				

Sweden	CG: Compression bandage (2 week)	2) NS (mean volume reduction, body weight, pain/heaviness/tension),% volume decrease favored IG (11% vs. 4%; p = 0.04)		

Pinell	IG: CDT (MLD + bandaging; MLD modified for patients with axillary or inguinal disease)	1) Limb volume	39 month	NR

2007 [56]				

U.S.	CG: As above (no modified MLD)	2) NS		

*BMSC *Bone Marrow Stromal Cell Transplantation; *CDT*: Complex Decongestive Therapy; *CG*: Control Group; *DASH*: Disability of Arm Shoulder and Hand; *hr *hour(s); *IG *Intervention Group; *IPC *Intermittent Pneumatic Compression; *LLLT *Low-level Laser Therapy; *MLD *Manual Lymph Drainage; *mo*: month(s); *NR *Not Reported; *NS *No Statistically Significant Difference Between Groups; *QoL *Quality of Life; *RCT *Randomized Control Trial; *ROM *Range of Motion; *SE *Secondary Lymphedema; *SF-36 Short Form *36; *SLD *Simple Lymphatic Drainage; *Tx *Treatment; *wk *week(s); *yr *year(s).

### Methodological quality assessment

Of the 36 English-language studies, 30 were RCTs [11,21-41,43-50] and six were observational (cohort) [51-56]. Fifteen RCTs were fair quality [21,22,25,26,29-32,36,43-47,50], eight were good quality [11,24,27,35,38,41,48,49], and seven were poor quality [23,28,33,34,37,39,40]. Among the observational studies, three were good quality [52,54,55] and three were poor quality [51,53,56].

The major quality issues with the RCTs were inadequate description of randomization processes in about half the studies, no reports of double blinding in a majority of the studies, and no discussion of methods to assess harms in most studies.

For the observational studies, the major quality issue was related to confounding. Four of the six studies [52,54-56] did not report attempts to control confounding. The authors of two studies [51,53] controlled potential confounding by matching on SE severity.

### Summary of extracted studies

Thirty-two of 36 English-language studies included participants with lymphedema secondary to breast cancer [11,21,22,24-32,34-41,43-52,54,55]. Some studies specified that participants had to be in remission, have no relapse, or have no metastases [21,22,24,26,29,35,41,43,44,46-48]. Five studies defined SE as 'mild' [21,22], 'chronic' [47], or 'moderate to severe' [24,48]. Sample sizes ranged from eight [32] to 150 [51].

Intervals between study participants' completion of cancer treatment and recruitment into the extracted studies varied considerably, e.g., 3 to 6 weeks [36], at least 3 months [46], at least 4 months [21,29], at least 6 months [22,27], at least 12 months [24,43,44], between 1 month and 1 year [47], or at least 4 years [30]. We also found variation in elapsed times between SE symptom onset and study recruitment, e.g., at least 3 months [32,34], greater than 3 months [50], a median of 9 to 10.5 months [31], less than 1 year [26], less than or equal to 2 years [25], or 0 to 5 years [49].

Follow-up periods varied considerably between studies, with little relation between follow-up length, study type, or intervention. Many studies ended immediately after the treatment regimen, although five studies followed patients for up to 1 year [34,41,51,53,54]. The shortest study lasted 24 h [24].

Several RCTs did not clearly label treatments as 'comparator' or 'experimental' (e.g., a study of IPC and MLD [31]). For this review, we assumed the comparators were the more conservative therapies. Common conservative therapies in RCTs were "usual care", sham treatment, or no treatment [11,26,27,32,36,37,43,44,52]. 'Active' treatment comparators included complex decongestive therapy [28,46,47], elastic sleeve [21,22,52], self-massage [49], bandaging alone [24,38], "simple lymphatic drainage" [45,50], IPC [34], MLD [23,30], or physiotherapy [33].

In the observational studies, comparators included IPC, compression garment, MLD, or no active treatment [51-56].

Many RCTs measured outcomes using limb volume or circumference [22,26,32,34,36,40]. Other outcomes included subjective symptoms such as pain, heaviness, or tension [28,30-32,34,36,55], range of joint motion (usually shoulder) [11,21,29-32,46], grip strength [31,34], measurements of intra- and extra-cellular fluid levels through bioimpedance [11,27], skin-fold thickness [43,44], and skin tonicity using tonometry [11,46,47]. Some studies attempted to correlate results of SE treatment with changes in quality of life [37,49].

For the observational studies, outcomes included limb volume [51-53,55,56], skin firmness [51], subjective assessments of body weight [55], limb circumference [56], and a vaguely described scale of 'psychic well-being' and 'physical complaints' [54].

### How effective are conservative treatments for SE in pediatric or adult populations who developed SE following any type of illness except filariasis infection?

Two RCTs showed IPC had benefits over CDT or self-massage [46,49]. Three other RCTs failed to show superiority of IPC compared to lymphatic massage [31], skin care [26], or elastic sleeve [22]. One RCT showed that a three-chamber IPC sleeve was better at reducing edema than a one-chamber sleeve [39].

Six RCTs used some form of massage-based therapy as the study treatment. Of these, only one suggested benefits in the massage group [25]. Other studies found no differences between massage and bandaging alone [38], elastic sleeve [21], or a less intensive form of massage [45,50].

In three studies of laser treatment, laser was superior to exercise [36], sham laser [11], or no treatment [35]. In a fourth laser study, laser was beneficial versus sham laser at intermediate time points [not at the endpoint], although the study authors did not provide quantitative statistical comparisons of the intermediate data [32].

Authors reported conflicting dieting results. One study showed no improvement with low fat or low caloric diets [44], while another showed improvement when dietary advice supplemented use of elastic sleeves [44].

Poor quality trials were more likely to suggest treatment benefits in experimental groups. Two RCTs involving IPC reported significantly more reductions in arm circumference when compared to MLD [40] or laser [34]. A study of bone marrow stromal cell transplantation versus decongestive therapy reported greater reductions in excess arm volumes with transplant (i.e., 81% vs. 55%; p < 0.001) [28].

The six observational studies examined a mixed group of treatments and found equivocal results: ultrasound was no different than IPC in reducing arm circumference [51], modified MLD reduced SE volume by 22% relative to standard MLD (authors did not report p-values) [56], group talks and exercise sessions added to MLD and compression stockings improved 'psychic well-being' (p < 0.05) yet made no difference in physical complaints [54], and persons with Kaposi's sarcoma who wore daily compression stockings had reductions in limb volume versus persons who wore no stockings (p < 0.001; authors failed to report the size of the treatment effect) [53]. Persons receiving MLD in addition to compression bandaging experienced less pain than persons receiving bandaging alone (p < 0.03), but the results showed no statistically significant reductions in absolute limb volume (p = 0.07) [55]. The final observational study compared sleeve to IPC and the authors found no significant differences in volume reductions between groups (the authors did not provide quantitative data) [52].

Some studies showed a loss of benefit by the end of the follow-up period. One observational study of elastic sleeve versus IPC found that both groups had returned to baseline levels within 4 to 12 weeks post-treatment [52]. Another study suggested a superior response to laser compared with sham treatment at 3 weeks following the last laser treatment. This benefit was lost after 7 weeks [32].

Considering the chronicity of SE, very few studies had long-term follow-ups. Eight of 36 studies reported outcomes at 6 months or more, with benefits shown to last for up to 1 year in some cases, usually with concomitant use of maintenance therapy (e.g., elastic sleeve).

### What harms are associated with conservative treatments for SE?

Harms were sporadically reported in the extracted studies. Only 17 of 30 RCTs reported harms [11,23-27,32-34,36,38,43-47,49]. The majority of harms were related to disease recurrence, not SE.

Some studies mentioned specific harms from therapy. These harms were rare, occurring in less than 1% of patients. Harms included infection, dermatitis [11,38], arm thrombosis [11,44], headache with elevated blood pressure [46], and arm pain [38]. None of these harms had major clinical impacts in any of the studies.

Only two studies compared harms between treatments. In an RCT evaluating bandages, subjects getting high-pressure bandages reported more pain and discomfort than subjects getting low pressure bandages, although the harms were measured using an invalidated scale [24]. A similar scale was used in an RCT comparing kinesiology tape with short stretch bandaging: subjects in the kinesiology tape group reported greater wound development than subjects in the bandage group (p = 0.013) [48].

No studies reported on factors that may increase the risk of harms associated with treatment.

### Non english-language studies

We included eight non-English-language studies. All eight studies were observational and involved breast cancer survivors with upper limb SE. Sample sizes ranged from 30 [57,58] to 440 [59]. Lengths of follow-up, where reported, ranged from 28 days [57] to 10 years [59].

Three studies examined single modality treatments: self-administered MLD versus an unspecified comparator, with improved arm function in the MLD group [60]; MLD delivered via the 'Asdonk standard' method versus 'non-Asdonk MLD', with greater reductions in arm volume in the Asdonk group (the authors described the Asdonk method, but did not reference the method, nor did they provide quantitative statistics or p-values) [57]; and single- versus multi-chamber IPC, with no differences in SE severity between groups at the end of follow-up [61].

Three studies investigated multi-modal treatments: multi-layer bandaging and MLD versus simplified bandaging and MLD, with larger decreases in edema occurring in the simplified bandaging group [62]; MLD, IPC, and exercise in two groups, with bandage added to one group, but no intergroup comparisons [58]; and IPC, IPC plus muscle electrostimulation, IPC plus magnetic therapy, or IPC plus both electrostimulation and magnetic therapy, with the largest percent change in limb volume occurring in the last group (p < 0.05) [59].

Two studies examined whether the time of treatment initiation affected outcomes. The first study compared treatment initiated within 1 year of breast cancer surgery to initiation within 1 or 2 years. Treatment in both groups was a combination of MLD, IPC, bandage, and exercise. Faster reduction of arm swelling was observed in the group with earlier treatment initiation [63]. Conversely, the second study found no differences between groups when treatment was initiated 3 months versus 12 months following SE diagnosis. The treatment regimen in this study was physical therapy, electrostimulation, massage, and IPC [64].

The non-English-language studies mirrored the high degree of heterogeneity observed in the English-language studies, e.g., different treatment combinations, varying lengths of follow-up. This heterogeneity prevented us from drawing clear conclusions to answer the key questions. The non-English articles did not contain substantive new information to supplement or alter our English-language findings.

## Discussion

Most extracted studies were conducted in persons with a history of breast cancer. One must be prudent before generalizing these studies' results to persons with other conditions.

Many studies showed that most active treatments reduced the size of lymphatic limbs, although extensive study heterogeneity in areas such as length of follow-up, treatment protocols, comparators, and outcome measures prevented us from assessing whether any one treatment was superior. The extracted studies did not contain reports of treatment benefits in any subgroup of patients.

Harms were reported in a small number of studies. These harms were rare and mild, and unlikely to be major clinical issues.

The methodological quality of the extracted studies was generally 'fair'. The authors of some studies omitted the reporting of fundamental elements of their research, such as the blinding of outcome assessors. Quality did not generally affect our interpretation of answers to the key questions.

### Research recommendations

Treatment protocols should be clearly described in published RCT reports (describing the comparator as 'usual care' is insufficient). If researchers believe a priori that important subgroup effects are possible, then the study should be powered to detect effects in these subgroups. Since a multiplicity of outcomes exists in SE research, researchers should develop a short list of preferred study outcomes. This will facilitate between-study comparisons and help make meta analyses feasible.

Experimental and comparator treatments must be clearly labeled and the comparator should be a standard treatment regimen for SE. Although sham treatments (e.g., laser) may satisfy minimum regulatory requirements for showing effectiveness, the clinical utility of a novel treatment is best demonstrated against an accepted standard treatment. Maintenance therapies, where used, should be clearly described by study authors. Blinding of study participants, clinicians, and healthcare professionals who administer treatment may not be possible due to the nature of the therapies; however, at a minimum, researchers should blind outcome assessors to treatment.

To avoid the publication of ambiguous trial reports, study authors should use existing quality scales [17-19,65] and the 2010 CONSORT statement for RCTs [66] as templates for producing RCT manuscripts. One of the extracted studies provides a good example of reporting an RCT's results [41].

Most of the extracted studies involved SE to the upper extremities. Few studies involved lower limb SE, despite its high incidence from cancer treatment [4]. More RCTs should be conducted in persons with SE of the lower limbs.

Another issue concerns whether treatment for the condition preceding SE would affect outcomes of conservative therapy for SE. For example, would patients treated with radiation therapy for breast cancer respond better to MLD than patients treated with lymphadenectomy? Research into this area could provide evidence to guide selection of SE therapy.

## Conclusions

Scientists have conducted a great deal of research into the treatment of SE. However, the literature contains no evidence to suggest the most effective treatment. Harms from treatment are minor and likely to have little clinical impact. The field of research into treating SE is open to advancement and we hope this review will guide future research in the area.

## Competing interests

The authors declare that they have no competing interests.

## Authors' contributions

All authors participated in the conception and design of the study. MO and ID summarized the extracted data. MO wrote the manuscript with contributions from all authors. All authors read and approved the final manuscript.

## Pre-publication history

The pre-publication history for this paper can be accessed here:

http://www.biomedcentral.com/1471-2407/12/6/prepub

## Supplementary Material

Additional file 1 **Methods S1**. Literature search strategies. Click here for file
