# Supplementary material for: Systematic review: conservative treatments for secondary lymphedema
Source: BMC Cancer. 2012 Jan 4;12:6. doi: 10.1186/1471-2407-12-6 (PMC3320521; doi:10.1186/1471-2407-12-6)
Supplement: Additional file 1 — Methods S1. Literature search strategies. [file 1471-2407-12-6-S1.DOC]

**Additional File 1, Methods S1**

**Literature Search Strategies**

Ovid MEDLINE(R)

Search Strategy:

--------------------------------------------------------------------------------

1 lymphedema/ or elephantiasis/

2 lymph?edema.tw.

3 elephantiasis.tw. not (elephantiasis, filarial/ or filarial.tw.)

4 (comple* adj (lymph?edema or lymphatic or decongestive) adj (therapy or physiotherapy or physical therapy)).tw.

5 manual lymphatic drainage.tw.

6 foldi.tw.

7 vodder.tw.

8 casley-smith.tw.

9 Intermittent Pneumatic Compression Devices/ not exp *thrombosis/

10 intermittent pneumatic compression.tw. not exp *thrombosis/

11 or/1-10

12 limit 11 to humans

13 limit 12 to yr="1990 -Current"

14 (comment or editorial or letter).pt.

15 13 not 14

Ovid EMBASE

Search Strategy:

--------------------------------------------------------------------------------

1 lymphedema/ or elephantiasis/

2 lymph?edema.tw.

3 lymph?edema.tw.

4 elephantiasis.tw. not (elephantiasis, filarial/ or filarial.tw.)

5 (comple* adj (lymph?edema or lymphatic or decongestive) adj (therapy or physiotherapy or physical therapy)).tw.

6 manual lymphatic drainage.tw.

7 foldi.tw.

8 vodder.tw.

9 casley-smith.tw.

10 Intermittent Pneumatic Compression Devices/ not exp *thrombosis/

11 intermittent pneumatic compression.tw. not exp *thrombosis/

12 or/1-11

13 limit 12 to human

14 limit 13 to yr="1990 -Current"

15 (editorial or letter or note).pt.

16 14 not 15

Ovid AMED (Allied and Complementary Medicine)

Search Strategy:

--------------------------------------------------------------------------------

1 lymphedema/

2 lymph?edema.tw.

3 elephantiasis.tw.

4 (comple* adj (lymph?edema or lymphatic or decongestive) adj (therapy or physiotherapy or physical therapy)).tw.

5 manual lymphatic drainage.tw.

6 foldi.tw.

7 vodder.tw.

8 casley-smith.tw.

9 pneumatic compression/

10 intermittent pneumatic compression.tw.

11 or/1-10

12 limit 11 to yr="1990 -Current"

Ovid MEDLINE(R) In-Process & Other Non-Indexed Citations

Search Strategy:

--------------------------------------------------------------------------------

1 lymphedema/ or elephantiasis/

2 lymph?edema.tw.

3 elephantiasis.tw. not (elephantiasis, filarial/ or filarial.tw.)

4 (comple* adj (lymph?edema or lymphatic or decongestive) adj (therapy or physiotherapy or physical therapy)).tw.

5 manual lymphatic drainage.tw.

6 foldi.tw.

7 vodder.tw

8 casley-smith.tw.

9 Intermittent Pneumatic Compression Devices/ not exp *thrombosis/

10 intermittent pneumatic compression.tw. not exp *thrombosis/

11 or/1-10

EBM Reviews: Cochrane Central Register of Controlled Trials

Search Strategy

1. lymphedema/ or elephantiasis/

2. lymph?edema.tw.

3. elephantiasis.tw. not (elephantiasis, filarial/ or filarial.tw.)

4. (comple* adj (lymph?edema or lymphatic or decongestive) adj (therapy or physiotherapy or physical therapy)).tw.

5. manual lymphatic drainage.tw.

6. foldi.tw.

7. vodder.tw.

8. casley-smith.tw.

9. Intermittent Pneumatic Compression Devices/ not exp *thrombosis/

10. intermittent pneumatic compression.tw. not exp *thrombosis/

11. or/1-10

12. limit 11 to yr="1990 -Current"

EBSCO CINAHL

Search Strategy

--------------------------------------------------------------------------------

S1 ( ("lymphedema") or (MH "Lymphedema") or (MH "Elephantiasis") ) or TX manual lymphatic drainage or TX foldi or TX vodder or TX casley-smith

S2 TX intermittent pneumatic compression not TI thrombo*

S3 TX complex lymphedema therapy or TX complex lymphatic therapy or TX complex decongestive therapy

S4 TX complete lymphedema therapy or TX complete lymphatic therapy or TX complete decongestive therapy

S5 TX complete lymphoedema therapy or TX complex lymphoedema therapy

S6 TX complete lymphoedema physiotherapy or TX complex lymphoedema physiotherapy

S7 TX complete lymphoedema physical therapy or TX complex lymphoedema physical therapy

S8 TX complex decongestive physiotherapy

S9 TX complex decongestive physical therapy

S10 TX complete decongestive physical therapy

S11 TX complete decongestive physiotherapy

S12 TX complete lymphatic physiotherapy or TX complex lymphatic physiotherapy

S13 TX complete lymphatic physical therapy or TX complex lymphatic physical therapy

S14 S1 or S2 or S3 or S4 or S5 or S6 or S7 or S8 or S9 or S10 or S11 or S12 or S13

S15 (MH "Compression Therapy")

S16 S14 or S15   limit Publication Year 1990-2010
